# Monitoring protein stability *in vivo*

**DOI:** 10.1186/1475-2859-4-23

**Published:** 2005-08-24

**Authors:** Zoya Ignatova

**Affiliations:** 1 Biotechnologie II, Technische Universität Hamburg-Harburg, Denickestr. 15, 21073 Hamburg, Germany

## Abstract

Reduced protein stability *in vivo *is a prerequisite to aggregation. While this is merely a nuisance factor in recombinant protein production, it holds a serious impact for man. This review focuses on specific approaches to selectively determine the solubility and/or stability of a target protein within the complex cellular environment using different detection techniques. Noninvasive techniques mapping folding/misfolding events on a fast time scale can be used to unravel the complexity and dynamics of the protein aggregation process and factors altering protein solubility *in vivo*. The development of approaches to screen for folding and solubility *in vivo *should facilitate the identification of potential components that improve protein solubility and/or modulate misfolding and aggregation and may provide a therapeutic benefit.

## Introduction

The protein folding problem has challenged scientists for more than three decades. The process of folding has been studied extensively in the test tube, commonly carried out at low protein concentrations in order to minimize any parallel off-pathway aggregation processes. The environment, however, that a newly synthesized protein encounters in the cell is far from these idealized *in vitro *conditions. The intracellular space presents a highly crowded limited volume predicted to favor association processes [26]. Given the potential for intermolecular interactions with other cellular components, the kinetic and thermodynamic parameters of folding/unfolding processes in the cell may be quite different from those ideal *in vitro *solutions. The complex intracellular environment significantly affects thermodynamic stability through changes in the local osmolarity and oxidation potential in response to stress conditions [3,25]. Reduced protein stability is detrimental to function and can lead to aggregation or degradation [2].

There are multiple instances where low *in vivo *stability of a protein can lead to negative outcomes. Recombinant protein production faces difficulties producing some proteins in an active form in expression hosts. Unfortunately, many recombinant proteins are not efficiently expressed in a folded or soluble form, which is an obstacle for the pharmaceutical industry and biotechnology. Protein-folding diseases are yet another area where protein solubility plays an important role. Aberrant folding and stability in the cell is associated with the pathology of many neurodegenerative diseases (e.g., Alzheimer's, Huntington's, Parkinson's disease) [2,16]. Solubility and folding efficiency of a protein can be maintained by sequence alterations, which necessitate a significant structural knowledge about the protein of interest to choose appropriate amino acids for mutation. As a result, there is an urgent need to develop methods to accurately monitor folding and unfolding events on fast timescales, directly in living cells. Such efficient selective methods to monitor folding and/or solubility *in vivo *could be also used to assay for compounds inhibiting aggregation and screening for variants with an improved solubility by natural or induced mutations.

## In-Cell Spectroscopy

The potential of the nuclear magnetic resonance (NMR) technique, widely used *in vivo *for identification of small molecules, has been extended towards determination of the conformation of proteins inside living cells [10,17]. The chemical shifts as a specific fingerprint of the local environment of the atomic nuclei within a protein provide residue specific information on the conformational changes due to unfolding or binding events, or other post-translational modifications. Studies using [^15^N,^1^H]-HSQC [18,19] or magic angle spinning binding experiments [10] have been used to characterize cytoplasmic and periplasmic biomolecules in bacterial strains. The *in vivo *NMR-spectra detect additional conformations not observable in *in vitro *purified samples: The in-cell spectrum of calmodulin reveals different occupation of the four calcium binding sites that is connected to the tight regulation of the intracellular calcium concentration [19]. A crucial factor, however, that limits the application of the in-cell NMR experiments is the rotational correlation time or tumbling rate of the protein in the cellular environment. The intracellular space is highly viscous and decreases the tumbling rate, causing a broadening of the peaks or even their disappearance. The introduction of the TROSY approach [13] has increased the molecular weight range of proteins that are amenable to NMR spectroscopy beyond the 100 kDa mark. This can be applied to virtually any protein, but there is still the viscosity limit of the intracellular space.

An interesting twist using conventional mass spectrometry technique, widely applied to the determination of conformational states of proteins, is a method to measure the Stability of Unpurified proteins from rates of H/D exchange (SUPREX) developed by Oas and coworkers [5]. This approach enables the determination of protein stability directly in the cell. This technique is a variant of the Matrix-assisted laser desorption/ionization (MALDI) mass spectrometry [4] and determines the protein stability *in vivo *by measuring the hydrogens that exchange with solvent deuterons at a rate depending on the thermodynamic stability of the protein [5]. The SUPREX approach has been applied to estimate the folding thermodynamics by direct in-cell urea titration. The thermodynamic stability of the small monomeric N-terminal fragment of λ repressor (λ_6–85_) estimated with this technique shows no change in stability versus measurements made *in vitro *[5]. Additionally, the effects of introduced mutations can be quantified as changes of the free energy (ΔΔ*G*^o^_N-M_) between the native and unfolded states of mutated protein (Δ*G*^o^_M_) compared to the wild-type counterpart (Δ*G*^o^_N_). The bacterial cells are viable up to 3 M urea, which is sufficient to map the entire unfolding transition curves *in vivo*. Although some successful examples to determine conformational changes and stabilities have been reported so far, both techniques, NMR and mass spectrometry, are far from being established as routine tests for estimating the *in vivo *stability. A serious limitation arises if the target protein interacts with any cellular components (e.g., chaperones, DNA, membranes).

## Protein-Reporter-Based Approach

**Figure 1:**
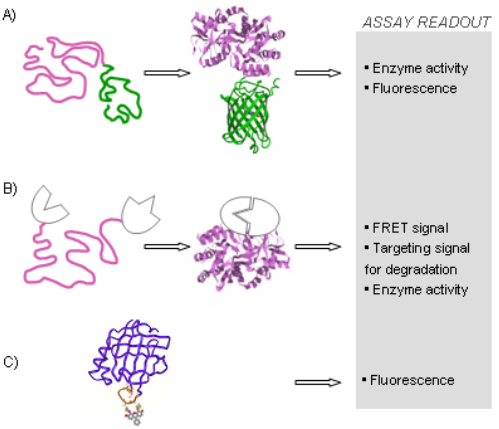
Strategies for site-specific labeling of proteins to detect folding and stability *in vivo*. (A) The autofluorescent green protein is appended to the protein of interest (here maltose-binding protein) and the latter dictates the spectroscopic behavior of the protein-reporter tag. The same fusion scheme applies for enzymes (e.g., CAT) whose activity serves as readout of the analysis. (B) Complementation assay. The target protein (here maltose-binding protein) is sandwiched between two reporter proteins or between the two halves of one reporter. Efficient folding brings both parts of the reporter unit into sufficient proximity to generate a readout signal. (C) Small-molecule labeling using peptide tags. The peptide tag can be introduced in the middle of the protein sequence (here cellular retinoic acid binding protein with a tetracysteine motif highlighted in yellow and FlAsH ligated to it) and the intensity of the emitted fluorescent signal can be used to determine conformationally distinct populations, provided the specific signatures of the folded and unfolded states are pre-determined for each experiment.

The fusion of a protein of interest to a reporter protein whose function depends on the solubility of the chimera has been successfully used to reveal *in vivo *protein stability and folding efficiency, provided the reporter does not change the solubility of the target protein [Fig. 1 is a cartoon summarizing different tagging approaches]. Here, the term solubility is used interchangeably with folding, and it denotes both the chemical solubility of the folded native state and the absence of aggregation due to misfolding events. This technique is based on the attachment of a specific protein tag that equips the resulting chimera with a unique property that can be exploited to monitor certain functions of the target protein *in vivo*. Davidson and coworkers used the resistance against chloramphenicol generated by fusing chloramphenicol acetyltransferase (CAT, 25 kDa) to the C-terminus of the protein of interest to select soluble variants from a large pool of mutants created by a random mutagenesis [11].

A specific implementation of the reporter-based approach is the structural complementation that involves the splitting of the reporter protein into two components that need to combine to execute their function. Wigley et al. [23] have designed an elegant assay for monitoring of the solubility/folding based on complementarity of two fragments originating from β-galactosidase. The α-fragment of β-galactosidase, typically 50–90 residues in length, when attached C-terminally to the protein of interest, can complement its ω-fragment, coexpressed in the soluble cytoplasmic fraction, to reconstitute the functional protein. The assembly occurs only if the target protein is expressed in the soluble form and the complementation event restores the enzymatic functions. The relatively small size of the α-peptide does not perturb the structure of the target proteins and does not have any dramatic effect on the solubility of the chimera [23]. As a bimolecular reaction the assembly is significantly affected by the concentration of the complementing parts and is determined by the equilibrium association/dissociation constant *K*_d_, which is a significant hurdle to study kinetics of folding/aggregation. An advantage this system shares with the CAT-based system is the potential for using an easily detectable phenotypic selection for screening of mutants with improved folding efficiency.

An alternative to the complementarity principle is the split-technique by which target protein is tagged at both termini with two halves of the reporter protein [9]. The protein of interest is sandwiched between the two halves of ubiquitin, which assemble into a functional ubiquitin moiety only after an accomplished folding of the target protein [15]. The reconstituted ubiquitin serves as a substrate of ubiquitin-specific proteases, normally present in the cytosol and the nucleus of all eukaryotic cells, and the release of the reporter from the chimera is the readout of the assay. Detection is accomplished *ex vivo *after the intermolecular association event has taken place. It may perturb the equilibrium established within the cell due to this physical separation of the cellular components. The method is qualitative, allowing the study of alterations of the structure caused by mutations or intracellular environment but it cannot be used for measurements and for determination of the destabilization energy (ΔΔ*G*^o^_N-M_) caused by the corresponding mutation.

The use of autofluorescent proteins as reporter protein tags, such as green fluorescent protein (GFP) and its relatives with improved spectral properties, has contributed greatly to our understanding of intracellular trafficking, localization and protein-protein interactions that could not have been achieved with other technologies [20]. The autocatalytically-generated broad fluorescent signal of GFP is easily detectable in a bulk cell suspension or under the microscope, and this key feature makes it an attractive fusion counterpart for studying intracellular folding and unfolding processes. Waldo and coworkers [21] showed that the folding trajectory of a protein of interest fused upstream to the GFP dictates its fluorescent behavior and the emitted fluorescent signal is directly proportional to the amount of the correctly folded protein. Later on, Glockshuber and coworkers [14] propose an approach for monitoring of protein solubility based on *in vivo *Förster-resonance-energy-transfer (FRET) measurements of two proteins, reminiscent of the idea of the complementation assay. The target protein is labeled at both termini with GFP and its blue-shifted variant, BFP, and efficient folding brings both fluorescent proteins into sufficient proximity to generate a FRET-signal. The readouts of the fluorescent reporter protein-based methods for protein solubility *in vivo *are simply detectable signals and allow a fast selection of the soluble variants of any protein of interest. Although enhanced variants of GFP with significantly accelerated folding and fluorescence acquisition have been developed, unfortunately, their bulky size (238 amino acids) may perturb the structure of the target protein. The relatively long folding trajectory of GFP itself ranging from minutes to hours [6], even of the improved Venus or T-Sapphire variants, is out of the time window of early intramolecular (folding) and intermolecular (aggregation) events. Furthermore, large surface contacts between the target protein molecules (e.g. protein aggregation) can undesirably alter the fluorescence of the reporter [24].

All reporter-based approaches monitor the protein folding and the solubility as a result of efficient folding at its equilibrium. For understanding the mechanisms of protein misfolding in the cell and its impact for disease and biotechnology, it is necessary to follow the process from its early initial steps. Insertion of the reporter molecule within the sequence of the target protein can probably capture early folding/unfolding events. Unfortunately, the bulky reporter proteins can be appended only to either the N- or C-terminus. Short peptide sequences that mediate the labeling of the protein of interest with small synthetic molecules, conferring unique spectroscopic properties, present an alternative solution to overcome these restrictions. Such approaches are discussed in the next section.

## Lighting-up Folding Processes with Small Molecules

The prototype for the *in vivo *labeling of fusion proteins with small synthetic molecules is based on the formation of stable complexes between biarsenical derivatives of fluorescein (FlAsH) or resorufin (ReAsH) and peptides containing tetracysteine motif developed in the lab of Roger Tsien [1,7]. The two arsenoid groups of the dye selectively coordinate the four cysteine residues from the naturally uncommon tetracysteine motif (Cys-Cys-Xaa-Xaa-Cys-Cys, where Xaa denotes any amino acid, preferably Pro-Gly, [1]), which can be incorporated at virtually any place in the target protein sequence due to the small size. The dye ligand is membrane permeable and non-fluorescent; however it emits an intense fluorescent signal upon high affinity binding to the cysteine residues (2–4 pM *K*_d _[1]). The background fluorescence can be minimized by treatment of the cells with ethane dithiol that pairs the unpaired cysteines from endogenous proteins displaying a weak affinity for FlAsH.

Dye ligation to the tetracysteine receptor is not connected to a characteristic emission shift that can be used as a specific fingerprint for detection of a distinct conformational species. Incorporation of the tetracysteine sequence in flexible structural regions (e.g., loops) within the protein sequence of the cellular retinoic acid binding protein, however, leads to a significant sensitivity of the dye quantum yield on the conformational state of the host protein, with a denatured state hyperfluorescent compared to the native one [8]. The FlAsH-mediated fluorescence lacks specific quantum yields repeatedly observed for distinct conformational populations and the frame conditions (e.g., intensity of folded and unfolded state) need to be determined for each protein. Upon a set-up of these frame conditions the intensity of the FlAsH fluorescence can be used to capture early misfolding events and to monitor the time course of aggregate formation, provided the FlAsH has been internalized in the cells before induction of the protein synthesis [8]. The key advantage of this system is that it allows to follow the whole protein folding history, starting from the early misfolding events assumed to generate the most toxic species in the aggregation process. Moreover, the FlAsH-mediated fluorescence can be used to monitor the equilibrium stability of a protein of interest by direct in-cell urea titration [8], allowing thus to determine the direct effect of a mutation on the protein stability and the aggregation propensity of mutants.

Recent improvements of the original FlAsH-methodology have expanded the utility of this approach, providing a powerful tool for studying conformational dynamics *in vivo*. Nakanishi and coworkers [12] modified the bensoic acid moiety and developed another biarsenical analog of the Nile red (BarNile) with an environmentally sensitive fluorophore used to monitor conformational changes caused by binding of metal ions. The FlAsH-based techniques hold a great promise as a tool for study the protein misfolding and aggregation kinetics in the cell with a time window tracking the early aggregation phases.

## Conclusions and Future Perspectives

Off-pathway misfolding and aggregation are highly undesirable processes both reflecting the cell's fitness, resulting in physiological dysfunctions (e.g., neurodegeneration), and the production of recombinant proteins. *In vivo *investigations, in the same background the newly synthesized protein encounters, are tremendously helpful to elucidate the mechanisms of protein folding/misfolding. The general need, however, goes towards detecting soluble and insoluble species after the biosynthesis process has reached equilibrium. Besides the development of methods with easy readouts, the approaches have to be conceptually focused on spanning the time window towards the early folding/unfolding events. Real-time kinetic studies are necessary to track the entire process and they will accelerate the development of strategies to suppress the undesired off-pathway unfolding reactions. In addition to understanding the mechanisms of folding and aggregation within the cell, the direct monitoring of the protein solubility *in vivo *has wide practical applicability. For instance, in the recombinant production of proteins, culture conditions can be modulated and optimized by monitoring their direct effect on the protein solubility. Of particular interest would be to engineer expression hosts, e.g., use of bacterial strains deficient in certain intracellular proteases, or strains with up-regulated expression of cellular chaperones and to follow in real time their influence on the recombinant protein production. The direct monitoring of the folding ability *in vivo *opens up the possibility of high-throughput screening for the production of proteins with improved solubility by random mutagenesis and facilitating the design of novel protein structures.
